# Dynamics of laser produced plasma from foam targets for future nanolithography devices and X-ray sources

**DOI:** 10.1038/s41598-021-93193-w

**Published:** 2021-07-01

**Authors:** Yaoxing Wu, Girik Jain, Tatyana Sizyuk, Xinbing Wang, Ahmed Hassanein

**Affiliations:** 1 Wuhan National Laboratory for Optoelectronics, Huazhong University of Science and Technology, Wuhan, 430074 China; 2 Center for Materials Under Extreme Environment (CMUXE), Purdue University, West Lafayette, IN 47907 USA

**Keywords:** Nanoscience and technology, Optics and photonics, Physics

## Abstract

Foam targets are expected to be more efficient candidates than solid targets for laser produced plasma (LPP) for extreme ultraviolet (EUV) and X-ray radiation sources due to the expected plasma conditions that can be optimized regarding plasma opacities, volumetrics heating effects, and the produced ions debris characteristics. In this paper, a comparison of ion dynamics between low-density foam and solid Ni plasma was systematically investigated at CMUXE. The foam Ni target (density 0.6 g/cm^3^) and solid Ni target (density 8.9 g/cm^3^) were irradiated with 1064 nm Nd:YAG laser in vacuum. A Faraday cup (FC) was used to record the ion flux and time-of-flight (TOF) signals. A lower and wider TOF signal was observed for foam Ni plasma on the time scale. The average ion energy and peak of the TOF signal of solid Ni plasma were much higher than that of the foam Ni plasma. However, the total charge values between foam and solid Ni plasma were comparable indicating a more volumetric absorption of laser energy for foam Ni. The average ion energy and peak of the TOF signal of solid Ni showed a stronger angular and laser energy dependence than that of foam Ni. The plume shape of the solid Ni plasma appeared as an oblong ellipse at each time, while that of foam Ni plasma tended to be more circular, especially at early times. The results of mass ablation rate were consistent with the FC signals and showed a more intense plasma shielding for solid Ni.

## Introduction

Laser produced plasma (LPP) with a high-power laser pulse opens up a new path for the development of innovative extreme ultraviolet (EUV) and X-ray sources which are very important for wide-ranging applications. These include advanced lithography^1^, X-ray backlighting^2^, inertial confinement fusion (ICF)^3^, Thomson scattering^4^, high energy density physics (HEDP)^5^, radiography^6^, etc. In these applications, it is crucial to acquire the EUV and X-ray sources with a high brightness and high conversion efficiency (CE) of laser to desired EUV or X-ray radiation output. The EUV and X-ray yield is determined by the plasma state which is significantly dependent on irradiation conditions such as laser intensity, pulse duration, focusing spot size, waveform, laser wavelength, etc.^7–12^. Furthermore, the target parameters (atomic number, structure, density, geometric shape, thermal conductivity, heat capacity, etc.) will also influence the final CE and an efficient source can be achieved by optimizing these parameters^13–18^. Currently, one important issue for the interaction between laser and solid targets is that the latter is highly opaque, thus limiting the interaction region to the skin layer of the target surface. Furthermore, the electron density of the plasma produced by the leading edge of the laser will reach the critical density quickly because of the high-density of the solid target, which causes a significant fraction of the laser energy to be reflected and a rather low laser absorption rate^19,20^. However, reducing the initial density of target used in LPP could effectively solve this issue^21^. Recently, the interaction between the laser and low-density foam targets is becoming a research hotspot.

When a pulsed laser is incident upon a low-density foam target, the laser is able to penetrate and heat the target directly to a much greater depth/volume and a controlled, low-density plasma will be generated, showing the so-called volumetric heating effect and relatively uniform density and temperature compared with solid targets^22^. These characteristics of foam targets can increase the laser absorption rate and reduce the ion kinetic energy, which is very beneficial for the EUV or X-ray conversion devices^23^. In the field of semiconductor lithography, liquid tin droplets irradiated by a double pulse laser scheme is currently adopted to enhance the EUV emission intensity with 100 kHz repetition^24,25^. However, the double pulse scheme has a durability problem due to the difficulty of controlling the droplet expansion dynamics and fragmentation caused by the pre-pulse^22,26^. Currently, some research related to the laser produced low-density tin plasma show that the complex double pulse laser scheme is expected to be replaced by using low-density foam targets in the future^26–28^. In addition, low density foam targets have been demonstrated to have higher X-ray radiation than solid targets in reports of the laser produced Au^23,29,30^, Cu^31^, V^31^, Ti^32^, Ca^33^, Bi^34^, and cellulose triacetate^20^ plasma. Thus, low density foam targets are expected to be the most suitable candidates for the efficient LPP EUV and X-ray sources^20^.

Currently, most of studies related to low density foam targets in LPP focus on their EUV or X-ray radiation. A related study^28^ compared the EUV spectral purity and ion debris mitigation between the low-density and solid Sn targets by using a transverse magnetic field. The results show that the ion flux can be effectively reduced more than 1 order of magnitude for the low-density Sn targets by using a 0.64 T magnetic field, while the magnetic field slowed down only Sn ions for solid Sn plasma. The unresolved transition array (UTA) spectrum at 13.5 nm showed a narrower distribution for low-density Sn plasma. Yunsong et al.^30^ studied the detailed energy distributions in laser produced solid and foam gold plasma by using a 1D hydrodynamic code. The calculated results show that the foam gold targets have stronger X-ray radiation and lower kinetic energy of ions, which is ascribed to the larger X-ray emission zone and smaller conduction zone. Fazeli^33^ simulated the effects of target porosity on the X-ray line emission from laser produced Ca plasma in the water-window region for different laser pulses with different intensities and durations. The results calculated with 1D Lagrangian laser plasma code also indicated that the X-ray lines were enhanced considerably for low-density Ca targets and the plasma ion density can be turned by increasing the target porosity. Lu et al.^35^ reported the plasma expansion with gold foam irradiated by laser. The theoretical analysis and MULTI 1D simulation results show that the density contour of gold foam moved slower than that of solid gold. Kaur et al.^20,29,36^ investigated the interaction of high-power sub nanosecond laser pulses with low-density cellulose triacetate polymer and gold foam targets. They demonstrated that the low-density foam targets in LPP could enhance the X-ray yield and reduce the kinetic energy of ions. Although many papers show that the X-ray radiation could be enhanced by using low density foam targets, further studies are still needed to fully understand the physics of laser interaction of low-density foam targets. Furthermore, although some papers report a decrease in ion kinetic energy for the low-density foam targets, the majority of these studies are theoretically calculated results obtained through some simulations. Per our knowledge, there are very few experimental studies on the comparison of ion dynamics, plasma plume expansion and mass ablation rate between the solid targets and low-density foam targets. Apart from CE, the ion diagnostic is also very important for the performance of LPP EUV and X-ray sources since the lifetime of collector optics in the systems is highly dependent on ions energy and flux. The energetic ions emitted from LPP will sputter the multilayered optics and significantly reduce their reflectivity^37^, resulting in frequent replacement of the expensive optical collector system. This damage to the collector optics is directly related to the ions kinetic energy. Ions with lower kinetic energy are preferable and beneficial for extending the lifetime of the collector optics. Currently, a multilayered ellipsoidal mirror is usually adopted to collect and focus the EUV or X-ray radiation. The detailed investigation of ion angular distribution characteristics not only provide a better understanding of the ion emission mechanism and the overall profile of the plasma expansion, but also provide guidance for the design of ellipsoidal collector optics. The determination of the ion dynamics, plasma plume expansion evolved with time and space and mass ablation rate dependence on laser energy could result in better understanding of the physics of laser interaction with low-density foam targets, which is critical for efficient and debris-free LPP EUV and X-ray sources.

In this paper, we have systematically studied and compared the characteristics of laser produced solid and low-density foam Ni plasma. The ions emitted from the LPP were collected by a Faraday cup (FC). The total charge, time-of-flight (TOF) signals and ion kinetic energy distributions of the LPP for solid and foam Ni plasma have been studied and compared in detail. In particular, the influence of laser intensity on these parameters and their angular dependence was also investigated. An intensified charge coupled device (ICCD) was used to capture the two-dimensional plasma plume images and a microbalance was adopted to calculate the mass ablation rate of the different targets. These experimental results could help in understanding the difference between laser interaction with solid and low-density foam targets.

## Experimental setup

**Figure 1:**
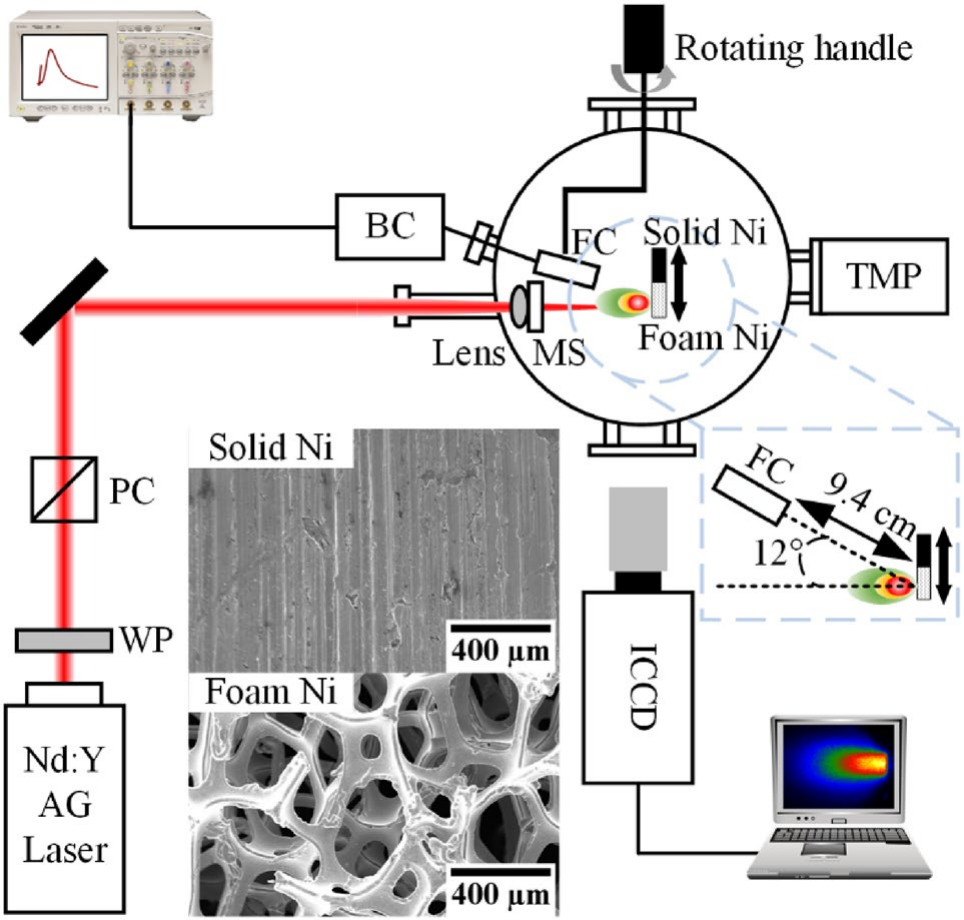
The schematic of the experimental setup in CMUXE and the pictures of solid and foam Ni under a scanning electron microscope. *BC* biasing circuit, *FC* Faraday cup, *MS* microscope slide, *TMP* turbomolecular pump, *PC* polarizing cube, *WP* half waveplate, *ICCD* intensified charged coupled device.

The schematic of the experimental setup in CMUXE is shown in Fig. 1. A Q-switched Nd:YAG (Continuum Surelite III) laser was used for generating plasma in a vacuum chamber and operated at the wavelength of 1064 nm. The full width half maximum (FWHM) pulse duration and unfocused circular beam diameter were 6 ns and 10 mm, respectively. A combination of a half waveplate and a polarizing cube was used to attenuate the output laser pulse energies. The laser energies were adjusted from 100 to 400 mJ in steps of 25 mJ to investigate the dependence of laser energy on ion dynamics. A 20 cm plano-convex lens was adopted to focus the laser beam onto the surface of the planar solid and foam Ni targets. A very thin microscope slide was connected to the lens to prevent the contamination coming from plasma debris and would be replaced after each 1000 pulses. The targets were placed in front of the lens focal point to obtain a larger spot size which was 520 μm. The peak power density of the laser for 100 mJ, 200 mJ, 300 mJ, and 400 mJ was 7.82 × 10^9^ W/cm^2^, 1.56 × 10^10^ W/cm^2^, 2.35 × 10^10^ W/cm^2^, and 3.13 × 10^10^ W/cm^2^, respectively.

The densities of the solid and foam Ni target were 8.9 g/cm^3^ and 0.6 g/cm^3^, with purities of 99.96% and 99.8%, respectively. The foam Ni target showed an open-cell network structure under a scanning electron microscope (SEM) which is shown in Fig. 1. Both of the solid and foam Ni were mounted side by side to a remotely controlled servomotor (ThorLabs Z825B) XYZ translation stage, which translated the targets around the laser irradiating position to provide fresh surface exposure for each measurement. The thicknesses of solid and foam Ni target were 1 mm and 3.2 mm, respectively. Silicon wafers were added to the back of solid Ni target to compensate for the difference in thickness. The position of laser focal point could also be adjusted on different areas of the target surface by the servomotor without opening the vacuum chamber, making sure that the experimental results of solid and foam Ni were obtained at the same conditions. The pressure in the vacuum chamber was kept at ~ 2 × 10^–5^ Torr during the experiment to minimize the charge exchange with the residual ambient gas.

A cylindrical Faraday cup (FC) ion collector (IC) with a 3 mm entrance aperture diameter was placed at a distance of 9.4 cm from the target surface to monitor the flux and kinetic energy of the ions induced by the laser-target interaction. The Faraday cup was mounted on a rotating handle with an angle meter which allowed the angle of measurement to be changed from outside the chamber. When the angle meter on the rotating handle showed 0°, it meant that the FC and the laser spot were on the same horizontal plane. At the time illustrated in Fig. 1, the angle between the FC and the target normal was 12°. A bias voltage of − 30 V was applied to collect the ions and to repel the electrons emitted from the plasma. The output signal was monitored and recorded using a 50 Ω load resistor 1 GHz oscilloscope (Agilent infiniium MSO8104A). Each ion signal was obtained with same experimental conditions by averaging the voltage signals 10 shots to minimize the influence of the fluctuation in the shot-to-shot laser energy. In order to minimize the contamination of hydrogen, carbon, and other contaminants, the Nd:YAG laser power was adjusted to a very low energy (~ 10 mJ, 1 Hz repetition) and keep the target moving to clean the target surface before recording the TOF signal. The cleaning process usually last about few minutes. After finishing this process, the laser was adjusted to ~ 100 mJ and operated in single-shot mode. Random locations on the target surface were chosen to compare the TOF signals’ difference between first shot and second shot. If there are no large difference between the first and the second shot, we assumed that the contamination has been removed and the experiments would start. The recorded TOF signals were therefore, assumed entirely due to the contribution of Ni ions.

For the evolution of plasma plumes, a series of time resolved plasma images were captured by an ICCD camera (Andor iStar DH334T-18U-E3 1024 × 1024) using a tele-photo objective lens (Edmund Optics 75 mm DG Series Fixed Focal Length Lens) through a quartz window in the vacuum chamber at a 90° angle with respect to the incident laser. The plasma images were composed of integrated radiation from a wavelength region of 180–850 nm. The gate width with 5 ns and 10 ns were used to image plasma at early times (< 100 ns) and at later times, respectively.

A microbalance (METTLER TOLEDO XS3DU, dual range balance with 1 μg readability) was used to measure the target mass before and after being irradiated by the pulsed laser. Two hundred shots were accumulated at different laser energies and the mass difference was used to calculate the mass ablation rate. All experiments were repeated 5 times and then take an average as the final results to improve the reliability of the data.

## Results and discussion

### Ion measurements

Understanding the ion emission characteristics and the effect of laser intensity on the ions’ kinetic energy and angular dependence are essential to investigate LPP evolution and dynamics.

**Figure 2:**
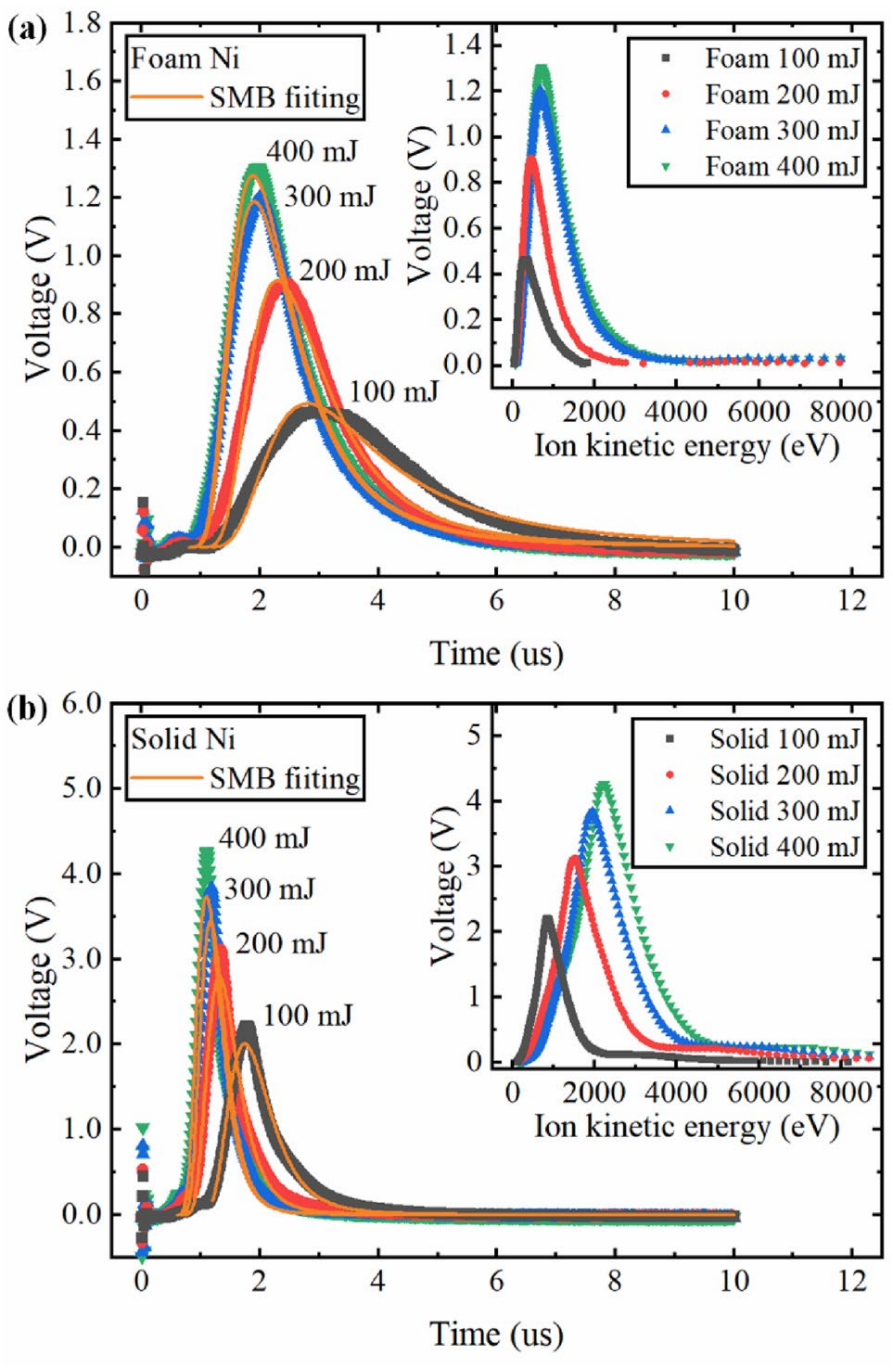
Typical ion time-of-flight (TOF) signal obtained from the Faraday cup ion collector for various laser energies for (**a**) Foam Ni and (**b**) Solid Ni. At the peak position, the percentages of error tolerance of voltage for foam Ni at 100 mJ, 200 mJ, 300 mJ, and 400 mJ are 15.3%, 15.7%, 10.5%, and 13.7%, and that for solid Ni are 4.1%, 5.7%, 4.4%, and 6.2%, respectively. The Shifted Maxwell Boltzmann distribution was used to fit the thermal peaks. The inset shows the ion kinetic energy distribution converted from the TOF signal.

**Figure 3:**
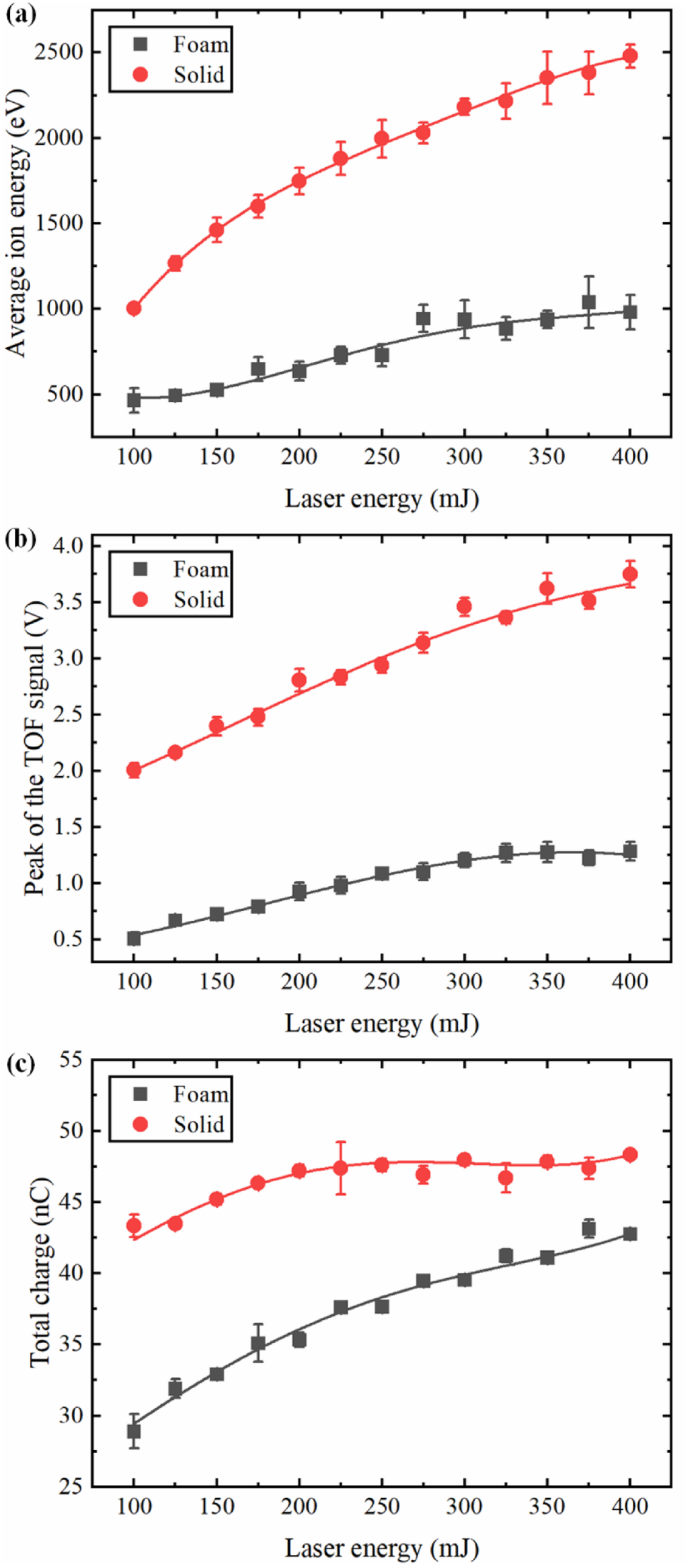
Effect of the laser energy on (**a**) average ion energy, (**b**) peak of the TOF signal and (**c**) total charge. The error bars represented the standard deviation derived from the data of five experiments.

Figure 2 shows a typical TOF signal obtained from the Faraday cup ion collector for various laser energies. Each curve was the average of the TOF signal for 10 consecutive laser shots. Considering that the voltages have different tolerances at different time, the percentage of the error tolerance ($\varepsilon _{{tolerance}}$) of voltage at the peak position was defined as:

1 $$ \varepsilon _{{tolerance}}  = \frac{{\max \left\{ {\left| {U_{n}  - U_{{ave}} } \right|} \right\}}}{{{1 \mathord{\left/ {\vphantom {1 2}} \right. \kern-\nulldelimiterspace} 2}\left( {U_{{ave}}  + U_{{\max }} } \right)}} \times 100\% , $$

where $U_{n}$ is the maximum voltage of each TOF signal, $U_{{ave}}$ is the average maximum voltage of 10 TOF signals, $U_{{\max }}$ is the maximum voltage of TOF signal when $\max \{ \left| {U_{n}  - U_{{ave}} } \right|\}$ among the 10 TOF signals. The percentage of error tolerance indicated that the TOF signals of foam Ni plasma had larger jitter compared with that of solid Ni plasma which is related to the network structure of foam Ni target. In general, the TOF signal of the foam and solid Ni all reached a peak quickly with time under different laser energies, and then decreased as the plume continued to expand beyond the FC. In addition, the rising rate of the TOF signal and its peak increased, and the position of ion peak shifted to earlier times as the laser energy increased, indicating an enhancement in the number of high-energy ions. In nanosecond LPP, the electrostatic model^38^ is usually used to explain the ion acceleration. The electrons will absorb the laser energy via inverse bremsstrahlung (IB) process and transfer the absorbed laser energy to ions through electron–ion collisions on the time scale of the electron–ion thermalization time (10^–10^–10^–11^ s) which is much shorter than the laser pulse duration. In other words, the electrons and ions reach equal thermal energies at the end of the laser. However, the electron velocities are much higher than that of the ions due to the huge mass difference. A space charge separation in the plasma plume is formed due to the huge velocity difference between the energetic electrons and the heavy ions, generating a self-electrostatic field. The ions are accelerated in this self-electrostatic field and the final ion kinetic energy is proportional to their charge state. However, this ion acceleration due to the generation of electrostatic field is more pronounced at higher laser intensities. Figure 2 shows such high energy ions detected at earlier time which are small fraction of the total ion kinetic energy. Previous experiments showed two peaks of ion kinetic energies in LPPs related to small fast ions fraction and large slow ions fraction^39^. HEIGHTS 3D simulations predicted very accurately the kinetic energies of the large ion fraction showing that most of kinetic ions in LPPs at the considered laser intensities can be explained by hydrodynamic effects in quasi neutral plasma plume^8,40^. Therefore, the increase of the average ion charge state as well as increasing plasma kinetic energy with the increase of laser energy is the most probably reason for the increase in ion kinetic energy of solid and foam Ni plasma. The increasing difference of average ion energy between solid and foam Ni plasma is shown in Fig. 3a and will be discussed later.

Generally, the LPP will experience complicated processes of ionization and recombination after it is generated. The TOF signal obtained from Faraday cup ion collector is composed of all ion energies and charge states emitted from LPP. Under the combination of ionization and recombination process, a shifted Maxwell–Boltzmann (SMB) is usually used to describe the ion kinetic energy distribution in LPP^38^:

2 $$ F\left( t \right) = C\left( {\frac{L}{t}} \right)^{3} \exp \left[ { - \frac{{m\left( {\frac{L}{t} - v_{{fit}} } \right)^{2} }}{{2k_{B} T_{x} }}} \right], $$

where $C$ is a scale parameter, $L$ is the distance between the FC and target surface, $v_{{fit}}$ is the center-of-mass velocity which is determined by the adiabatic expansion and the coulomb acceleration, $k_{B}$ is the Boltzmann constant, and $T_{x}$ is the translational temperature. According to the SMB fitting, the center-of-mass velocity of foam Ni plasma at 100 mJ, 200 mJ, 300 mJ, and 400 mJ were 1.62 × 10^4^ m/s, 2.88 × 10^4^ m/s, 3.39 × 10^4^ m/s, 3.40 × 10^4^ m/s, and that of solid Ni plasma were 4.66 × 10^4^ m/s, 6.22 × 10^4^ m/s, 7.22 × 10^4^ m/s, and 7.55 × 10^4^ m/s, respectively. These velocities provide guiding relevance for the plasma plume expansion. Based on the comparison of center-of-mass velocity between the foam and solid Ni plasma, we can also infer that the plasma plume expanded faster for solid Ni plasma along the target normal, which was also verified by the time-resolved plasma plume images in next section.

The shape of TOF signal highly depends on the ion density distribution in the plasma plume along the specific direction. The TOF signal of foam Ni plasma in Fig. 2 experienced a longer time and showed a much smaller amplitude than that of solid Ni plasma, indicating a lower and wider distribution for foam Ni plasma on the time scale. This meant that the ion density distribution in the foam Ni plasma plume was relatively more uniform compared to solid Ni plasma. Similar results are found in laser produced low-density polymer aerogels plasma^36^ and gold foams plasma^29^ studies. The inset in Fig. 2 shows the ion kinetic energy distribution converted from the TOF signal which provide a more intuitive comparison of the ion kinetic energy between the solid and foam Ni plasma. The proportion of high-kinetic energy ions in the solid Ni plasma was much higher than that of the foam Ni plasma. For example, in the case of the 400 mJ laser energy, there were almost no ions with kinetic energy greater than 2000 eV in the foam Ni plasma, while nearly half of the ions with kinetic energy greater than this value existed in the solid Ni plasma. This is very important in terms of the lifetime of the optical collecting system, e.g., in nanolithography devices. Lower energy ions of the foam targets will cause less damage than the energetic ions of the solid targets.

In order to further compare the plasma characteristics between the foam and solid Ni plasmas, the effect of the laser energy on the average ion energy, peak of the TOF signal, and the total charge is shown in Fig. 3. The average ion energy can be calculated as:

3 $$ \begin{aligned}    & \overline{E}  = 1/2M\left( {\frac{L}{{\overline{t} }}} \right)^{2}  \\     & \overline{t}  = {{\int_{{t_{i} }}^{{t_{f} }} {tV(t)dt} } \mathord{\left/ {\vphantom {{\int_{{t_{i} }}^{{t_{f} }} {tV(t)dt} } {\int_{{t_{i} }}^{{t_{f} }} {V(t)dt} ,}}} \right. \kern-\nulldelimiterspace} {\int_{{t_{i} }}^{{t_{f} }} {V(t)dt} ,}} \\  \end{aligned} $$

where $M$ is the mass of the ion, $t_{i}$ and $t_{f}$ respectively are the start and end time values of the ion signal, $V(t)$ is the voltage measured by the FC. As expected, the average ion energy of the solid and foam Ni plasma increased with the increase of laser energy, but the rising rate was slowly decreasing, and that of foam Ni plasma showed a saturation trend. The average ion energy of solid Ni plasma was greater than that of foam Ni plasma and the difference gradually increased with the increase of laser energy. For instance, the difference of the average ion energy between solid and foam Ni plasma was 536.4 eV at 100 mJ, and this value became 1498.7 eV at 400 mJ. These results implies that ions are thermalizing at lower kinetic energy and weak laser energy dependence in case of foam targets which is very important for the fusion research and EUV debris reducing where lower energy ions are preferable.

It is well known that the laser can only propagate in the plasma below the critical electron density. During the interaction between laser and plasma, an ionization wave (heat wave) and a rarefaction wave will be created in the conduction zone and the interface between the plasma and vacuum, respectively^20,23^. If the velocity of ionization wave propagating into the bulk target is higher than that of sound in the already heated matter, significant hydrodynamic motion will not be generated in spite of the very large pressure gradients at the position of heat front^20^. Various studies^20,23,30^ related to laser produced under-dense target (the ionized electron density lower than the laser critical density) plasma pointed out that the ionization wave generated by laser ablating under-dense target is supersonic and its velocity is inversely proportional to the target density^29,41^, while the rarefaction wave is subsonic. In the case of under-dense plasma, no hydrodynamic motion of the dense plasma occurs because of the supersonic laser heating, resulting in no hydrodynamic shock wave generated. Therefore, the absorbed laser energy will not be transferred to hydrodynamic motion. Alternatively, when the ionization wave front is close to or coincides with the rarefaction wave front, a considerable amount of laser energy will be converted to rarefaction wave inside the over-dense plasma plume which enhances the conversion of the laser energy to the ion kinetic energy^20,36,41^. The electron density $n_{e}$ of the target can be calculated from the mass density $\rho$ by^42^

4 $$ n_{e}  = \rho \frac{{\overline{Z} }}{{M_{{atom}} }}A_{v} , $$

where $\overline{Z}$ is the average ionization state, $M_{{atom}}$ is the average atomic weight, and $A_{v}$ is the Avogadro’s number. Although the electron density of the foam and solid Ni under absolute ionization (2.56 × 10^24^ cm^−3^ for solid Ni, 1.72 × 10^23^ cm^−3^ for foam Ni) were both greater than the critical density (1.03 × 10^21^ cm^−3^ for 1.064 μm laser), the ionization wave velocity in foam Ni plasma is relatively larger compared with solid Ni plasma due to the lower initial density of foam Ni. Although the ionization wave velocities in solid and foam Ni plasma are both subsonic, the larger ionization wave velocity in foam Ni plasma will cause a larger distance between the ionization wave front and rarefaction wave front, thereby reducing the fraction of laser energy converted to ion kinetic energy.

Figure 3b,c show the peak of the TOF signal and the total charge under different laser energies, respectively. The peak of the TOF signal reflects the maximum ion density of plasma plume in a specific direction and the total charge represents the ion yield during the interaction between the laser and targets. It is obvious that the two parameters of the solid Ni were greater than those of the foam Ni due to the huge difference in target density. The total charge was calculated by integrating the corresponding TOF signal shown in Fig. 2^43^:

5 $$ Q = \int_{{t_{i} }}^{{t_{f} }} {\frac{{V(t)dt}}{R}} , $$

where $R$ is the load resistance. It can be seen that the peak of the TOF signal and total charge increased with laser energies for foam and solid Ni. The peak of the TOF signal for foam and soldi Ni both increased about 2 times in the studied laser energy range. The rise of these two parameters with laser energies can be due to the following two reasons. First, laser with high-energy would ablate more target material and have a higher mass ablation rate. Second, the increased laser energy enhanced the plasma heating process by the IB mechanism and lead to a higher degree of ionization in the plasma^43,44^. By comparing the peak of TOF signal between the foam and solid Ni, it can be clearly seen that the peak of TOF signal of the solid Ni was about 4 times that of the foam Ni under each laser energy, which confirms that the maximum ion density in solid Ni plasma plume was much higher than that of foam Ni plasma. The total charge in Fig. 3c also shows a saturation behavior for solid Ni in the studied laser energy range, which was not obvious for foam Ni. The saturation behavior for solid Ni could be ascribed to the more intense plasma shielding and reflection at the critical density point, which was also verified by the mass ablation rate shown in “Mass ablation rate”. Similar saturation trend was found in laser ablation of other solid materials^39,45^. The reason of the difference between the solid and foam Ni plasma could be explained as follows. During the processes of nanosecond laser target interactions, once the critical density is reached, the rest of the laser energy will be reflected and absorbed at the edge of the plasma plume which is then used to increase the ion kinetic^46^. Generally, the radiation absorption length along the beam axis can be treated as the so-called geometrical transparency length $L_{T}$, which is determined by the classical collision mechanisms^20,36,41^:

6 $$ L_{T}  = \frac{{9.2 \times 10^{{ - 8}} }}{Z}\left( {\frac{A}{Z}} \right)^{2} \frac{{T^{{3/2}} }}{{\lambda ^{2} \rho ^{2} }}, $$

where $Z$ is the charge of the plasma ions,$A$ is the atomic number, $T$ is the electron temperature (keV), $\lambda$ is the wavelength of the laser (μm), and $\rho$ is the plasma density (g/cm^3^). From Eq. (6), it is clear that the geometrical transparency length for laser radiation is inversely proportional to the squared foam (plasma) density, which means that the geometrical transparency length is shorter for solid Ni target due to its higher initial density. Therefore, when the high-power laser irradiated the solid Ni target, the plasma generated by the leading edge of the laser pulse would reach the critical electron density faster than that of the foam target and most part of the absorbed laser energy only took place near the critical electron density point, resulting in a higher electron density gradient. Main part of the increased laser energy will be reflected at the critical density point in the case of the solid Ni, which prevented to ablate more target material. Due to the lower initial density of the foam Ni, the critical density point was deeper inside of the target and the laser can penetrate the target to a much greater depth^20,33^. In other words, the under-dense corona zone (the electron density lower than critical density) of the foam Ni plasma was larger than that of solid Ni plasma and the electron temperature and density in the foam Ni plasma plume were more uniform^22^. This phenomenon can also be reflected to a certain extent from the lower and wider TOF signal of the foam Ni shown in Fig. 2. These characteristics of foam Ni exhibited the so-called volumetric heating effect and a higher laser energy absorption as the laser energy increased^20,22,23,33^. Therefore, the saturation behavior of total charge was more obvious for solid Ni. The increase of the total charge for foam Ni plasma was much greater than that of solid Ni plasma in the studied laser energy range.

**Figure 4:**
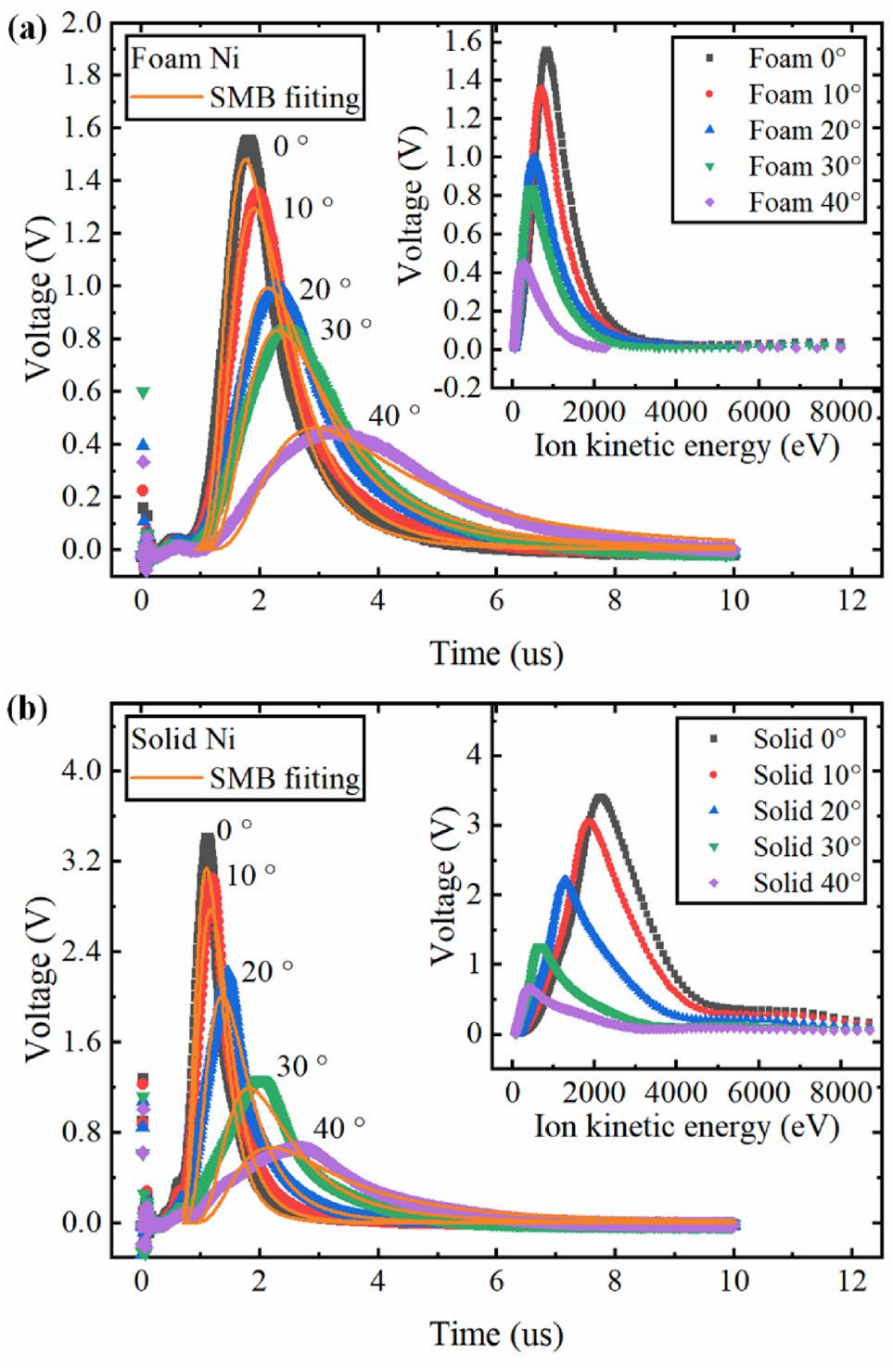
Typical ion TOF signal obtained from Faraday cup ion collector for various angular (**a**) Foam Ni and (**b**) Solid Ni. At the peak position, the percentages of error tolerance of voltage for foam Ni at 0°, 10°, 20°, 30° and 40° are 9.7%, 9.0%, 8.2%, 9.0%, and 15.4%, and that for solid Ni are 5.1%, 3.3%, 3.3%, 4.3%, and 3.4%, respectively. The 0° means that the FC and the laser spot were on the same horizontal plane. The Shifted Maxwell Boltzmann distribution was used to fit the thermal peaks. The inset shows the ion kinetic energy distribution converted from the TOF signal.

Understanding the angularly resolved distributions of the ion flux and kinetic energies are essential to study the mechanisms of ion production and plume expansion dynamics. For better understanding of the angular dependence of the ions emitted from foam and solid Ni plasma, the FC was mounted on a rotating handle with an angle meter and placed at various angles with respect to the horizontal plane, and the ion TOF signals were recorded and shown in Fig. 4 when the laser energy was 400 mJ. The solid lines given in Fig. 4 correspond to the SMB fitting using Eq. (2). It is clear that the peak of the TOF signals for solid and foam Ni occurred at later times and the amplitudes decreased with the angles. The TOF signal amplitude of the solid Ni was larger than that of the foam Ni at various angles and the TOF signal of the solid Ni had a narrower distribution than that of the foam Ni. The inset in Fig. 4 shows the ion kinetic energy distribution and the results illustrated that the maximum probable energy of the ions from both foam and solid Ni plasma decreased with an increase in off-normal angles. When the off-normal angles increased from 0° to 40°, the most probable ion kinetic energy of the solid Ni plasma dropped from 2120 to 400 eV, and that of foam Ni plasma dropped from 830 to 270 eV.

**Figure 5:**
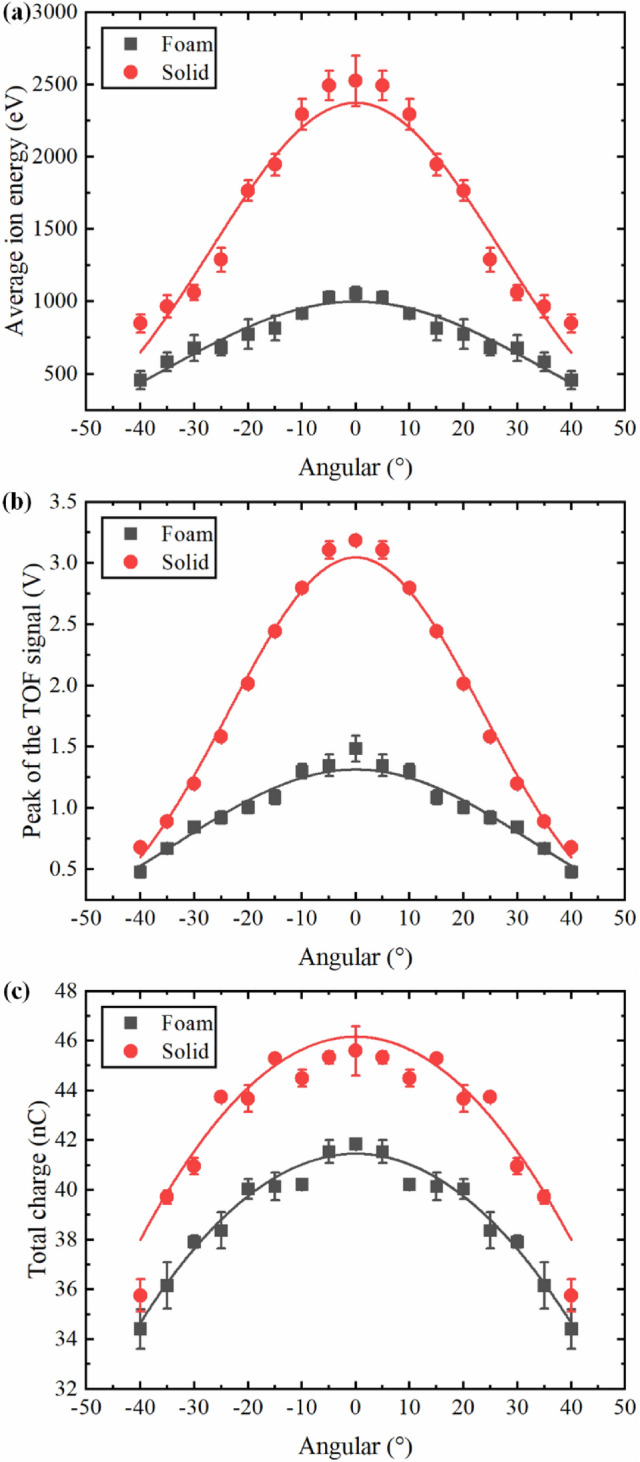
Angular dependence of (**a**) average ion energy, (**b**) peak of the TOF signal and (**c**) total charge. The error bars represented the standard deviation derived from the data of five experiments. The solid curves in each plot represent the ${\mathrm{cos}}^{n}\theta$ fitting to the various data sets.

In order to compare the angular dependence of the ions between the solid and foam Ni plasma, the average ion energy, peak of the TOF signal and total charge under different angles were calculated and shown in Fig. 5. The TOF signals were recorded for positive angles but copied to their negative angles for comparison assuming hemispherical expansion of the plasma plume. Generally, the angular distribution of the ions can be described by the $\cos ^{n} \theta$ function when a laser pulse is irradiated on a planar target in the normal direction^38^, where $n$ is typically larger than 1. The solid curves shown in Fig. 5 represent the $\cos ^{n} \theta$ fitting to the various data sets. The results show that the average ion energy, signal amplitudes, and the total charge strongly peaked when the angle was directly normal to the target and this behavior was more obvious for solid Ni plasma.

These three ion parameters of solid Ni were larger than that of foam Ni at various angles, but the difference between them gradually decreased with the increase of the angle. The average ion energy and peak of the TOF signal of solid Ni showed a stronger angular dependence than that of foam Ni. For example, the average ion energy and peak of the TOF signal of solid Ni were reduced to only 33.6% and 21.3% of the initial values at 40°, respectively. In the case of foam Ni, the average ion energy and peak of the TOF signal were reduced to 43.4% and 32.2% of the initial values at the same angle, respectively. Based on the fitting results, the exponent $n$ was estimated with an accuracy of 10% at 3.09 and 4.87 for foam and solid Ni in Fig. 5a, and at 3.41 and 6.11 in Fig. 5b, respectively. These results indicated that the plasma plume expansion was more isotropic for foam Ni compared with solid Ni, which could be ascribe to the larger geometrical transparency length and the volumetric heating effect. In the case of the total charge, both the solid Ni plasma and foam Ni plasma have similar angular distributions. Although the average ion energy and peak of the TOF signal of foam Ni plasma were much lower than that of solid Ni plasma, the total charge between the foam Ni target and solid Ni target was comparable in spite of the density of the foam Ni target was less than 1/10th of the solid Ni target. These results indicated that a large number of ions were coming from the large volume of foam Ni plasma and supported the more volumetric absorption of laser energy for low-density foam targets, which was also consistent with the laser produced low-density foam gold^29^, SiO_2_^47^ and polymer aerogels^36^ plasma studies and some simulations^30,33^.

### Plasma plumes

While the FC ion collector can provide interesting ion dynamic results along with the specific expansion direction, it is difficult to provide the two-dimensional shape of the plasma plume. In order to further investigate the plasma expansion dynamics, fast photography studies of foam Ni plasma and solid Ni plasma were carried out in order to record a series of time-resolved plasma plume images. These images provide the details of the LPP expansion dynamics, especially at very early stages after plasma generation.

**Figure 6:**
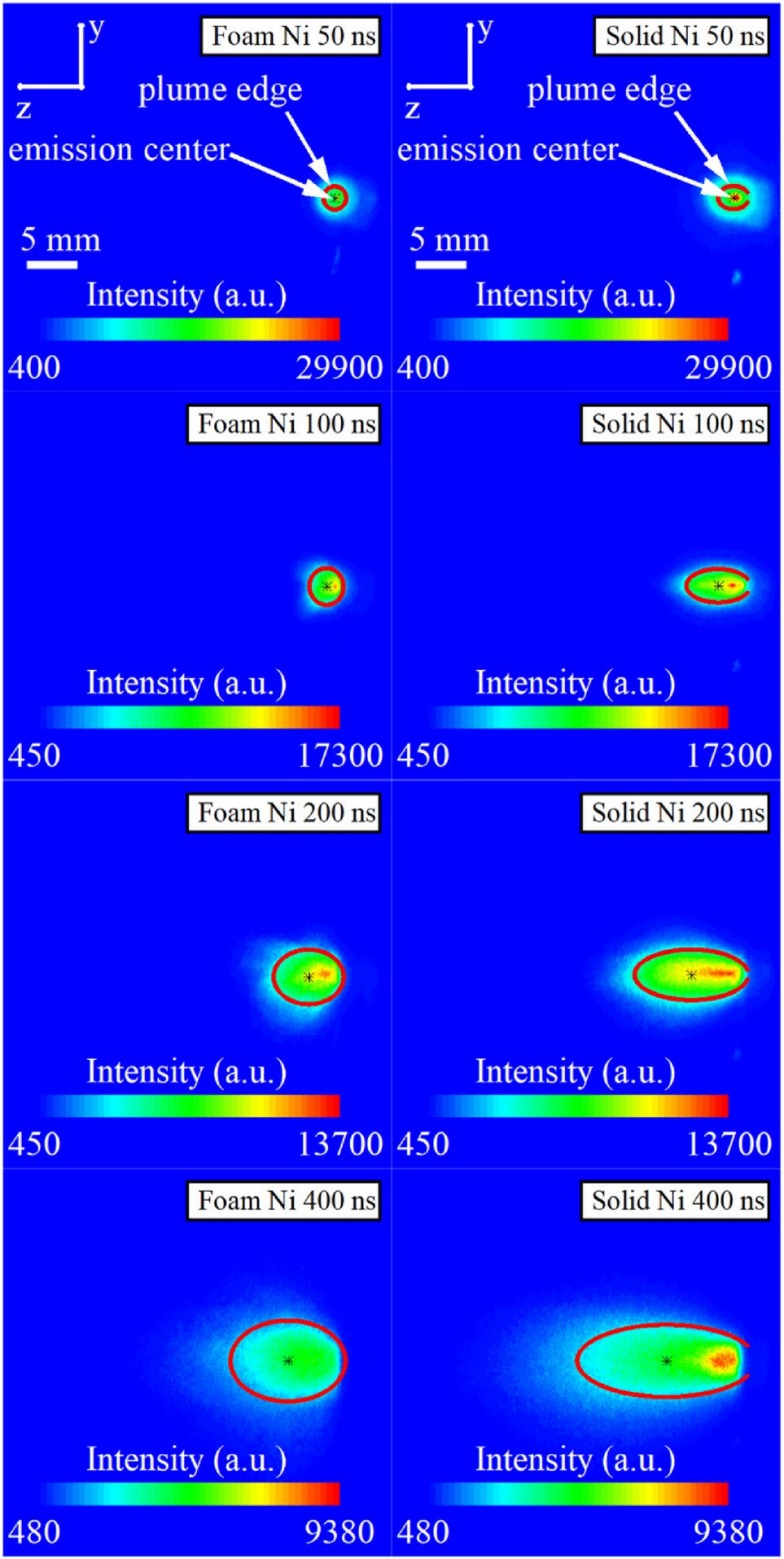
Time sequence of the two-dimensional ICCD images showing the spatial–temporal evolution of the plasma plume for foam and solid Ni. The laser irradiated on the targets along the z direction and its energy was 100 mJ. The black star (*) in each image represents the emission center determined by the 1st-order moment of the emission intensity. The red ellipse in each image represents the plasma plume edge determined by the 2nd-order moment of the emission intensity. Each image at particular time is normalized to maximum intensity with respect to solid Ni images.

Figure 6 gives the time sequence of the two-dimensional ICCD images showing the spatial–temporal evolution of the plasma plume for the foam and solid Ni. The laser energy used for this measurement was 100 mJ and the gate width of the ICCD was fixed to 5 ns at early times (< 100 ns) to minimize the spatial–temporal mixing and to optimize the imaging of internal structures. After 100 ns, the gate width was fixed to 10 ns to compensate for lower radiation at later times. Unlike the plasma plume expanding into an ambient gas condition, a sharp boundary was not observed in ICCD imaged plumes. In order to further investigate the plume expansion dynamics, determining the emission center and plume edge is very important and necessary. Based on the magnetohydrodynamics and self-similar expansion model^48^, the plasma plume generated by a laser irradiating a plane target can be approximated as an ellipsoid. Therefore, the plume shape of the LPP can be suitably described by an elliptic equation. In this study, the 1st-order moment and 2nd-order moment of the plume emission intensity were adopted to define the emission center and plume edge, respectively. The emission center was calculated as^49^:

7 $$ \overline{z}  = \frac{{\int_{{ - \infty }}^{{ + \infty }} {\int_{{ - \infty }}^{{ + \infty }} {zI\left( {z,y} \right)dzdy} } }}{{\int_{{ - \infty }}^{{ + \infty }} {\int_{{ - \infty }}^{{ + \infty }} {I\left( {z,y} \right)dzdy} } }},\overline{y}  = \frac{{\int_{{ - \infty }}^{{ + \infty }} {\int_{{ - \infty }}^{{ + \infty }} {yI\left( {z,y} \right)dydz} } }}{{\int_{{ - \infty }}^{{ + \infty }} {\int_{{ - \infty }}^{{ + \infty }} {I\left( {z,y} \right)dydz} } }}, $$

where $z$ is the direction along the laser irradiating, $y$ is the direction parallel to the target surface, and $I$ is the emission intensity of the plume. The semi-major axis and semi-minor axis of plume edge ellipse were calculated as^49^:

8 $$ \begin{aligned}    & R_{z}  = 2\sqrt {\sigma _{z}^{2} }  = 2\sqrt {\frac{{\int_{{ - \infty }}^{{ + \infty }} {\int_{{ - \infty }}^{{ + \infty }} {(z - \overline{z} )^{2} I\left( {z,y} \right)dzdy} } }}{{\int_{{ - \infty }}^{{ + \infty }} {\int_{{ - \infty }}^{{ + \infty }} {I\left( {z,y} \right)dzdy} } }}}  \\     & R_{y}  = 2\sqrt {\sigma _{y}^{2} }  = 2\sqrt {\frac{{\int_{{ - \infty }}^{{ + \infty }} {\int_{{ - \infty }}^{{ + \infty }} {(y - \overline{y} )^{2} I\left( {z,y} \right)dzdy} } }}{{\int_{{ - \infty }}^{{ + \infty }} {\int_{{ - \infty }}^{{ + \infty }} {I\left( {z,y} \right)dzdy} } }}} . \\  \end{aligned} $$

Generally, the plasma plume of foam and solid Ni both showed an almost symmetrical distribution with respect to the normal direction of the targets, and both expanded freely with little external viscous force because of the lower ambient pressure and the much higher initial pressure of the plasma. It is clear that the emission intensity of solid Ni was higher than that of foam Ni due to the higher initial density. The plume shape of the solid Ni plasma appeared as an oblong ellipse at each time, while that of foam Ni plasma tended to be more circular, especially at early times. By comparing the position of plume edge, it can be seen that the expansion velocity of solid Ni plasma was much higher than that of foam Ni plasma along the laser irradiating direction. This meant that the expansion dynamics of solid Ni plasma was more anisotropic than that of foam Ni plasma, which was consistent with the FC signal studies.

**Figure 7:**
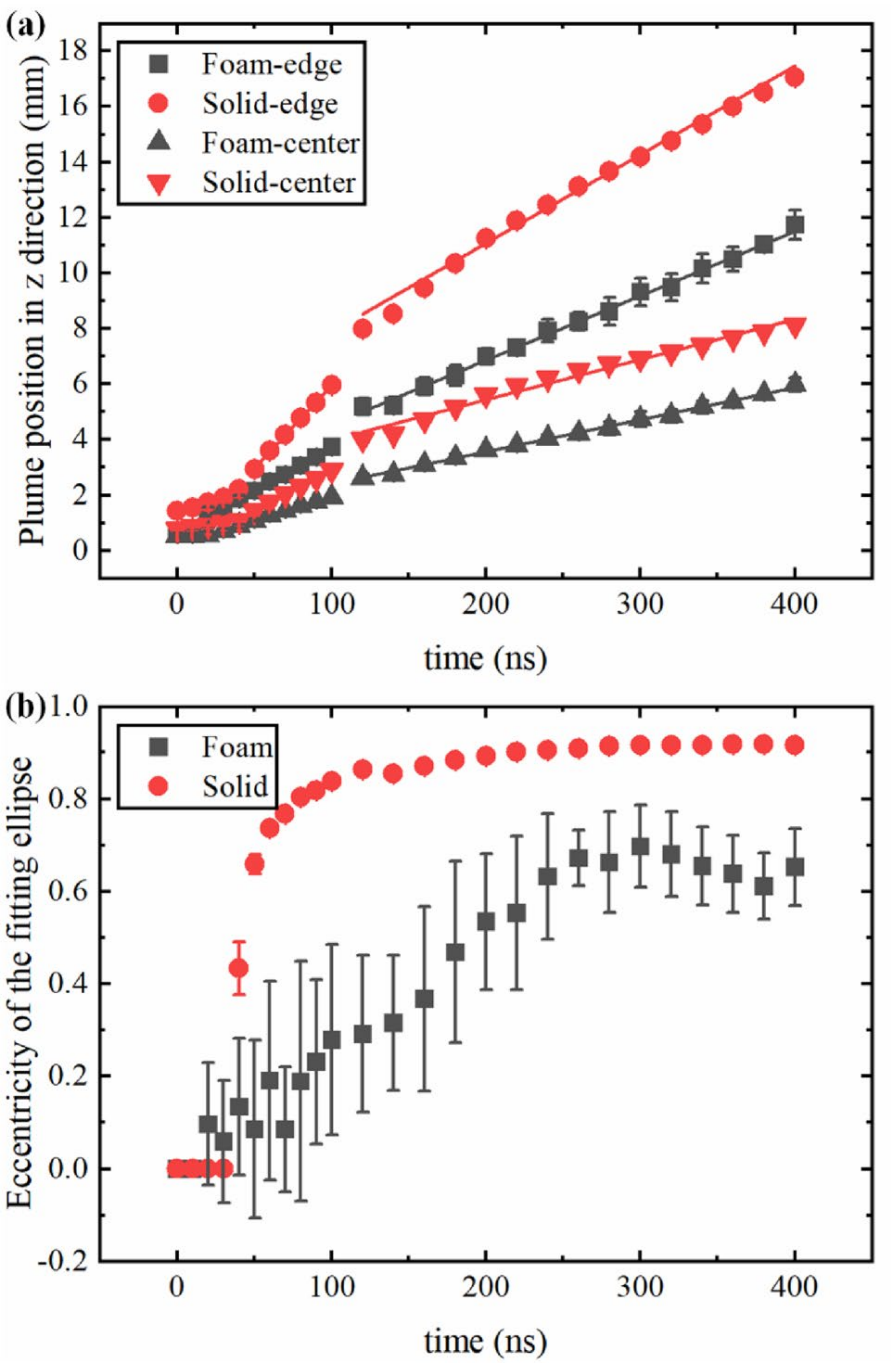
(**a**) Temporal variation of the plume position in z direction obtained from ICCD images. The ‘edge’ (black square, red circle) represents the position of fitting ellipse edge. The ‘center’ (black triangle, red inverted triangle) represents the position of the fitting ellipse center. The solid lines correspond to the linear fit. (**b**)Temporal variation of the eccentricity of the fitting ellipse. The error bars represented the standard deviation derived from the data of five experiments.

Figure 7 gives a more direct comparison of the plume position along the laser irradiating direction between the foam and solid Ni plasma as well as the eccentricity of the fitting ellipse. The results show a linear trend for the plasma plume of foam and solid Ni at later times which means that the plasma was expanding freely because the ambient conditions were almost near vacuum. This linear trend was more obvious for later times due to the less collisions. There was a jump in plume position between 100 and 120 ns because the gate width of ICCD was fixed from 5 to 10 ns. The average velocities of the plume edge derived from the plot for foam and solid Ni were 2.94 × 10^6^ cm/s and 4.27 × 10^6^ cm/s, and that of the emission center for foam and solid Ni were 1.49 × 10^6^ cm/s and 2.03 × 10^6^ cm/s, respectively. These results were comparable with the average velocity (3.45 × 10^6^ cm/s for foam Ni, 5.43 × 10^6^ cm/s for solid Ni) and center-of-mass velocity (1.79 × 10^6^ cm/s for foam Ni, 4.65 × 10^6^ cm/s for solid Ni) determined from the FC signal. The velocities derived from the ICCD images were smaller than that determined from FC signal and the reason could be as follows. The TOF signal recorded by Faraday cup was only contributed by ions in the plume. However, the plasma plume captured by the ICCD was recorded integrally from a wavelength range of 180–850 nm, which meant that the plume intensity included much radiation from neutrals. Nevertheless, according to the electrostatic model^38^, the neutrals cannot be accelerated in the self-electrostatic field and their velocities are much lower than ions in the plasma plume, which has been demonstrated by theoretical and experimental studies^38,50^. Therefore, the neutrals emission included in the plume would reduce the average velocity determined from the time resolved ICCD images.

Figure 7b shows the temporal variation of the eccentricity of the fitting ellipse. The eccentricity of the fitting ellipse could reflect the shape of the plume and be determined as:

9 $$ e = \sqrt {1 - \left( {\frac{{R_{y} }}{{R_{z} }}} \right)^{2} } , $$

where $R_{y}$ and $R_{z}$ are the semi-minor axis and semi-major axis of plume edge ellipse. The eccentricity of foam and solid Ni plasma plume was at 0 at very early times which meant that plasma plume shape was more like a circle, and then the eccentricity increased with time until a plateau was reached. The eccentricity of solid Ni plasma plume had a much higher increasing rate than that of foam Ni plasma plume and the final value of solid Ni plasma plume was higher than that of foam plasma plume. The results indicated that the plume shape gradually evolved from a circle to a flatter ellipse with time and the solid Ni plasma had a stronger angular dependence than that of foam Ni plasma. The larger eccentricity jitter of the foam Ni plasma plume could be due to the fact that the foam Ni target had a random network structure and was kept moving during the experiment, resulting in a slightly pressure difference in the plume produced by each laser shot. The eccentricity jitter of the foam Ni plasma plume was smaller at later times since the plume difference was compensated with plume expansion.

### Mass ablation rate

**Figure 8:**
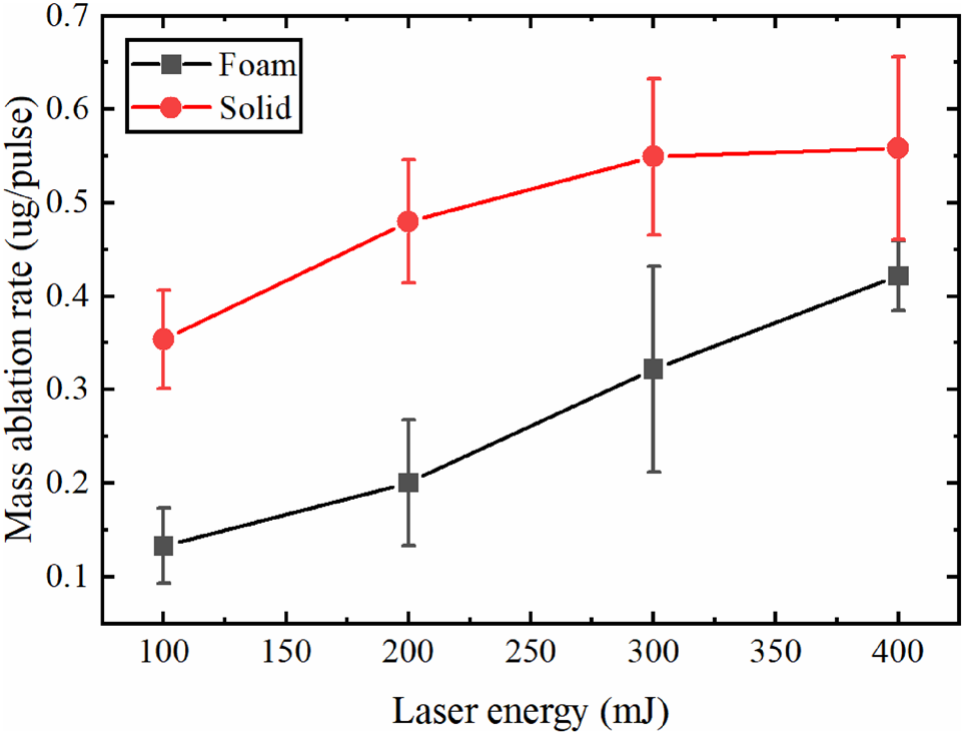
Trend of mass ablation rate for foam and solid Ni targets at various laser energies. The error bars represented the standard deviation derived from the data of five experiments.

Studying the mass ablation rate gives useful information about the interaction between laser and target. Figure 8 shows the trend of mass ablation rate for foam and solid Ni targets at various laser energies. The mass ablation rate of solid and foam Ni increased about 1.6 times and 3.2 times in the investigated laser energy range, respectively. Although the mass ablation rate of soldi Ni was higher than that of foam Ni at various laser energy, the values between them were somewhat comparable in spite of the huge difference in target density. In addition, a near saturation trend of mass ablation rate was found for the solid Ni which was absent for foam Ni in the investigated laser energy range. These results were consistent with the total charge shown in Fig. 3c and indicated that the plasma shielding effect was more intense for solid Ni.

## Conclusion

Foam targets are expected to be more efficient candidates than solid targets for laser produced plasma (LPP) for extreme ultraviolet (EUV) and X-ray radiation sources due to the expected plasma conditions that can be optimized regarding plasma opacities, volumetrics heating effects, and the produced ions debris characteristics. The ion emission from solid and foam Ni targets during 1064 nm wavelength Nd:YAG laser ablation was investigated in detail using Faraday cup (FC). The average ion energy, peak of the time-of-flight (TOF) signal, and the total charge was measured as a function of laser energy and off-normal angles. It was shown that the TOF signal of foam Ni plasma demonstrated a lower and wider distribution than that of solid Ni plasma on the time scale, which indicated that the ion density distribution in the foam Ni plasma plume was relatively more uniform compared to solid Ni plasma. The ion emission features at various angles showed a stronger angular dependence for solid Ni plasma. The average ion energy and peak of the TOF signal of foam Ni plasma were much lower than that of solid Ni plasma, but the total charge was comparable in spite of the huge difference in target density. These results supported the more volumetric absorption of laser energy in case of low-density foam targets due to the larger volume of the foam plasma below the critical density of the laser beam. The plasma plume images recorded by an intensified charge coupled device (ICCD) camera illustrated that the plasma plume of solid Ni plasma was more elongated along the laser irradiating direction compared with that of foam Ni plasma. The average velocities determined from time-resolved images was slightly smaller than the average velocities derived from FC signals, which was ascribe to the low-speed vapor and atom emission contained in plume. The mass ablation rate of solid Ni was higher than that of foam Ni, but the difference between them decreased with the increase of the laser energy, which indicated that the plasma shielding effect was more intense for solid Ni. The kinetic energy of ions produced from the foam Ni target was much lower than that of the solid Ni target. This is very important in terms of the lifetime of the optical collecting system, e.g., in nanolithography devices. Lower energy ions of the foam targets will cause much less damage than the energetic ions of the solid targets, therefore much longer lifetime.

## Data Availability

The datasets generated during and/or analyzed during the current study are available from the corresponding author on reasonable request.
